# Who benefits? Health equity and the Translational Science Benefits Model

**DOI:** 10.3389/fpubh.2025.1565248

**Published:** 2025-04-03

**Authors:** Anna La Manna, Julie Heidbreder, Shannon Casey, Kat Phelps, Mia LaBrier, Laura Brossart, Ella Clark, Emmanuel Tetteh, Sara Malone, Douglas A. Luke, Todd Combs

**Affiliations:** ^1^Center for Public Health Systems Science, Washington University in St. Louis, St. Louis, MI, United States; ^2^School of Medicine and Public Health, University of Wisconsin-Madison, Madison, WI, United States; ^3^School of Nursing, University of Wisconsin-Madison, Madison, WI, United States

**Keywords:** community engagement, translational science, health equity, impact evaluation, framework

## Abstract

**Introduction:**

Evaluating the impacts of translational science is crucial for demonstrating the quality, relevance, and societal benefits of research. This paper presents current results of efforts to expand the Translational Science Benefits Model (TSBM), a framework and toolkit originally developed at Washington University in St. Louis with 30 specific, real-world benefits across clinical, community, economic, and policy domains. In response to a growing emphasis on health and social equity, we have refined the TSBM to better address and integrate ideas of fairness and justice.

**Methods:**

Our methods included a literature scan to identify health equity gaps in the framework, community listening sessions in St. Louis, MO, and Madison, WI, and thematic analysis to incorporate equity into the TSBM.

**Results:**

The results introduce new dimensions within the existing TSBM domains that include 10 new benefits, all emphasizing themes of trust, power, and access.

**Discussion:**

Our aim is to enhance the relevance and utility of the framework and tools to researchers, practitioners, and those affected by implementations of findings from translational science and research. The integration of equity into the TSBM supports continued growth in the number of users and uses of the framework and toolkit to demonstrate health and social impact.

## Introduction

Evidence of the broader health, social, economic, and policy impacts from clinical and translational science is key in demonstrating the quality, usefulness, and relevance of empirical research to society. Individuals and communities meant to benefit from interventions, programs, or scientific discoveries, organizational staff and leadership, funders, and governments all have interests in downstream outcomes from science and research. Evaluation efforts – specifically those focused on impact – can demonstrate accountability from science and research to many different groups. More broadly, impact evaluation highlights the value that interventions create (1). Translational science and research exist to accelerate the realization of these impacts (2), from the development of research innovations through the implementation and sustainment. We define translational impacts of science and research as the advances that demonstrably increase health and well-being or health equity for individuals, families, communities, populations, regions, or systems. We conceptualize health equity as a continuous process of expanding the principles of fairness and justice in opportunities for all persons to achieve the best possible health outcomes.

The Translational Science Benefits Model (TSBM) is a framework for evaluating the downstream health and social impacts of clinical and translational research. It was created as part of the broader evaluation of the Institute of Clinical and Translational Sciences (ICTS) at Washington University in St. Louis (WashU). The ICTS is one of over 60 “hubs” awarded by the National Clinical and Translational Science Awardees (CTSAs) program at the NIH (3). First published in 2018, the TSBM originally included 30 specific benefits of translational science comprising four domains: clinical, community, economic, and policy (4). These translational benefits from science do not replace more traditional indicators of scientific contributions such as publication and research funding metrics but rather reflect further downstream impacts of science in society. The 30 benefits are available online at https://translationalsciencebenefits.wustl.edu/benefits/. Methods and strategies used to develop the TSBM framework are published elsewhere (4).

Our team continues to refine the framework and has developed the complementary *Translating for Impact Toolkit* to help scientists, programs, and institutions apply the TSBM to their work by integrating impact throughout the research process and intervention implementation (5). Individuals and groups can use any of this set of nine free web-based tools to plan, track, and demonstrate the impact of their work. Planning for impact tools include the *Road Map to Impact*, *Benefits 2×2, Pattern Mapper,* and *Team Manager* tools which help ensure that multiple and necessary points of view and areas of expertise are represented. The *Impact Tracker* helps to organize milestones toward specific benefits, and tools like the *Product Navigator, Case Study Builder, Impact Profile,* and *Dissemination Planner* help to demonstrate impact by conceptualizing, creating, and disseminating translational products for different purposes and audiences. Using the provided dissemination product templates and guides, teams can specify whether each benefit claimed is potential or demonstrated, allowing applications for projects at all phases, from projects in development to others that have formally ended. Multiple other CTSA hubs, along with other educational, scientific, and research institutions and programs across the US and internationally, use the TSBM framework and toolkit for planning, training, and evaluation.

While health equity has been studied for decades (6), major contemporary socio-cultural and health events, such as the COVID-19 pandemic, have brought to light the deeply entrenched inequities within communities and health systems around the world (7, 8). There has been increased attention on issues of social and health equity in all aspects of life, and a growing emphasis on health equity in research and practice reflects this (9–14). The recommendations of Healthy People 2030 included achieving health equity with the overarching goal to improve health and well-being for all (15). In line with these shifts, we systematically examined the extent to which the TSBM framework and toolkit clearly spoke to equity. TSBM case studies have demonstrated how several of the 30 benefits we originally identified can highlight increases in equity, for example improving *Healthcare Delivery* to better serve food-insecure communities of color (16), tailoring existing *Therapeutic Procedures* to better address drug use in students facing adversity (17), and developing and implementing new *Guidelines* for treating physical health risks of adults with serious mental illness in outpatient facilities (18). Additionally, all nine of the tools for TSBM have components that explicitly address equity considerations in research and implementation projects. That being said, there is much room for improvement in how the TSBM explicitly includes health equity.

Here we describe our approach to update the TSBM to clearly include explicit, community-vetted, health equity benefits and present current findings. For these efforts, our team includes the TSBM group from WashU and colleagues from the University of Wisconsin-Madison (UW) Institute for Clinical and Translational Research. We first explain our data collection and methodological strategies, followed by a presentation of proposed new benefits that focus on equity. We conclude with a discussion of how the updated TSBM can help scientists and organizations demonstrate the positive impact of their work on addressing health equity in society.

## Materials and methods

We set out to explicitly integrate health equity into the TSBM. We began by searching the scientific and gray literature to help identify gaps in the originally identified 30 benefits of the framework and engaged community members and groups from communities to gain different perspectives on health and healthcare. After synthesizing findings from all these efforts, we developed new benefits for the framework in an iterative process that included presenting and getting feedback from community groups and members. The new health equity benefits were presented to the ICTS Translational Research Program Officer, the ICTS Associate Director of Operations, and other members of the ICTS Evaluation team for review. The new health equity benefits were then presented to members of the ICTS External Advisory Board during an annual meeting. Currently, we are gathering additional feedback from groups of researchers and scientists to further study how these updated benefits can be applied in research and practice. As this work is ongoing, input from these researchers will be included in future papers.

### Literature scan of equity impacts

We conducted a literature scan to assess how equity impact is expressed and measured in health research and evaluation. Specifically, we searched for peer-reviewed articles and existing toolkits, frameworks, and other templates that included health equity in assessments of the broader impacts of science and research. We used a semi-structured approach to search PubMed, Google Scholar, and Olin PRIMO, a search tool that scans multiple databases developed at WashU. Key search terms included: health disparities, health equity, measurement, monitoring, social determinants of health, and surveillance. We also used the following combinations of terms: unjustness or discrimination or inequality or disparity or equity or inequity or equality or (social and determinant) plus health plus evaluation or indicator or measurement or monitoring or assessment or outcome. Additionally, we specifically searched journals in implementation science, translational science, public health, and evaluation (e.g., *Implementation Science*, *Clinical and Translational Science*, *American Journal of Evaluation*). Key concepts from the literature scan were compared to the original TSBM benefits to identify gaps and opportunities in the framework related to health equity. This comparison and its findings were the basis for discussion with community members.

### Community listening sessions

Near the end of the literature and TSBM reviews, we conducted a total of three community listening sessions. We recruited new and existing partners consisting of individuals and representatives of community groups with lived experiences of health and healthcare inequities from St. Louis, Missouri, and Madison, Wisconsin. In St. Louis, we invited community groups that were previously engaged with the ICTS at WashU as participants in *community studios*. Community studios are not focus groups, and therefore they do not collect demographic information. They serve to inform researchers with community or patient input for research development or implementation. For our community studio, participants were required to be community implementers of evidence-based programs and involved in policy and/or advocacy. For Madison, we recruited individuals from the Community Advisors on Research Design and Strategies (CARDS) Program, a group in existence for 15 years through the Wisconsin Network for Research Support (19). CARDS members were recruited by staff at local community centers as people who live in under-resourced neighborhoods and regularly used their services, such as food pantries, senior meals and educational programs. The CARDS group consists of 24 members with diverse demographic backgrounds. The majority of members (75%, *n* = 18) identify as Black or African American, while 16.7% (*n* = 4) identify as White, and 8.3% (*n* = 2) identify as Other. Of the 24 CARDS members, 66.7% (*n* = 16) identify as female and 33.3% (*n* = 8) identify as male. The age of CARDS members ranges from 23 to 81 years, with a mean age of 51 years. While direct income data is not collected, 50% (*n* = 12) of members self-identify as low-income, and 50% (*n* = 12) identify as not low-income. A total of 20.8% (*n* = 5) of members identify as having a disability, while 79.2% (*n* = 19) do not. Our goal for the sessions was to develop a better understanding of how to maximize the relevance and usefulness of equity considerations in the TSBM. We convened one session in St. Louis in person and two virtual sessions from Madison.

For the St. Louis session, we gave a brief presentation to introduce participants to the TSBM and summarize our literature scan findings. Prior to the session, we prepared three key questions to stimulate thinking about how the TSBM benefits could better recognize, describe, and demonstrate increases in health equity as translational impacts. The three questions were: (1) What is the one largest equity impact that your work has had?, (2) What equity benefits should we consider adding to the TSBM?, and (3) Take a look at the current TSBM benefits, are there any equity considerations that we should attend to? If so, what are they? We readied additional probes to elicit more conversation and details if needed and followed our discussion by asking about dissemination strategies (e.g., audiences, media and modes for sharing) and for overall reflections. This session was 90 min long and led by an expert facilitator. The WashU TSBM team provided an introduction, posed the questions to participants, and guided discussion as needed.

The input gathered from the St. Louis session was synthesized using an inductive thematic analysis approach. While formal transcripts were not created, detailed notes were taken during each session, capturing key discussion points, participant insights, and emergent themes. The research team reviewed these notes collectively to identify recurring patterns and concepts.

For the first Madison session, we repeated the presentation and revised the questions from insights gained during the first one in St. Louis. We wanted to more directly ask about and capture not only the direct lived experiences of participants but also their impressions about what equity looks like in their communities. The revised questions included: (1) What specific benefits are absolutely essential to you from health research or health care? and (2) How do you know the impacts have been fairly distributed so that all people can benefit from research? Both sessions from Madison were 90 min long. In the second session, we presented a set of proposed benefits developed iteratively after the St. Louis and first Madison session and solicited final feedback from participants.

Madison sessions were recorded, transcribed, and de-identified. Those transcripts were reviewed by members of the UW-Madison team for themes related to TSBM equity themes. In addition, team members aggregated their written notes with staff person notes taken during the meetings. After both Madison sessions, 10-page reports summarized outcomes and recommendations, including quotes that supported findings. While no special software was used, a reflexive thematic approach was used, with researchers generating themes through meaningful engagement with the data, the ability of themes to deepen with multiple reads of transcripts, and reflections upon our own experiences as researchers that brought assumptions and priorities into our work (20). Braun and Clarke (20) note six recursive phases: “familiarization; coding; generating initial themes; reviewing and developing themes; refining, defining and naming themes; and writing up (p. 39).

### Synthesis

Following the literature scan, TSBM review, and listening sessions, we compiled findings and continued refinement of the health equity benefits for the TSBM framework. To synthesize and organize key insights from the literature review and two community listening sessions, the team employed a digital collaboration tool, MURAL. This platform facilitated the structured visualization of diverse perspectives, allowing for the categorization and synthesis of themes and ideas. This approach supported the identification of key themes and facilitated a shared understanding among team members.

With these findings and through discussions, formal meetings, and email communications, we worked through several rounds of editing and feedback from the team and community collaborator colleagues to produce the benefits described here.

## Results

Many themes emerged from our efforts to explicitly include health equity considerations in the TSBM framework. We learned from existing literature and relevant materials how others formally describe, categorize, and operationalize concepts of equity. Community listening sessions provided opportunities to hear about the lived experiences of healthcare participants and users. We held the first session in St. Louis and the other two virtually for Madison. We hosted 8 community organization leaders in St. Louis and 8 and 14 CARDS (21) members in the first and second Madison sessions. Half (7) of the participants for the last session had attended the first one for Madison and the other half were new to the project but all were experienced CARDS members. Using the information gained through the literature scan and listening sessions we developed 10 additional benefits to update the original list of 30 published with the TSBM framework.

### Preliminary findings

In the literature scan, we identified and reviewed 58 peer-reviewed articles and four gray literature sources. Through reviewing the abstracts or introductions, 15 sources were selected for full review (Appendix A). We found multiple examples that fit directly into one of the 30 original TSBM benefits and could be easily added to the longer descriptions of existing benefits. For example, adding routine screenings for the social determinants of health under the Diagnostic procedures benefit or adding the removal of racialized or economic barriers to care under the Healthcare accessibility benefit.

Many of the themes we identified from existing sources, however, did not fit neatly into existing benefits, and we used them as a starting point to develop both the materials for the first listening session and initial sketches of potential new benefits. In the first listening session, the group shared general insights about increasing equity in healthcare and health outcomes and provided guidance on how to expand existing benefits. For example, they suggested including community members as deliverers of health education programs and developers of “Health education resources.” The group also identified the retention of diverse healthcare professionals as a potential benefit. Specifically, participants noted that healthcare providers from marginalized groups often experience racism in the workplace, which contributes to increased turnover. They suggested that addressing such racism could improve workplace culture and increase retention.

In the first session for Madison, community members followed up with explications of barriers to health and healthcare. In most cases, they cited scarcity or absence of essential resources or conditions and shared lived experiences with health and systems of healthcare. Among the missing or lacking components were transportation, access to quality care, insurance, affordable care options, and trust and understanding. Table 1 summarizes findings from the literature scan and the first two listening sessions, organized by the four TSBM domains (clinical, community, economic, policy). More quotations from the Madison session are available in Appendix B.

**Table 1:** Themes, insights, and quotations from existing sources and initial listening sessions.

	Themes from literature	Concepts from St. Louis session	Quotes from Madison (session 1)
Clinical	Data equity	Believe people’s own experiences Fair access to services regardless of clinical study participation Center patients in research process Practice shared decision-making Use understandable language	*“…within our community, it’s hard for people to trust health care providers. It’s hard for people to also understand what they are talking about.”* *“… with a lot of African American women, you know, we have been gaslighted.”*
Community	Built environment Community capacity Community engagement Education access and quality Health impact assessment Partnerships	Increase attention to building relationships Increase agency in people’s own health outcomes	*“…And there are people that miss appointments simply because it’s just like it’s too much of a struggle to have to load up your two kids on a bus…”* *“…they wanted me to go travel all the way to Milwaukee to get my tooth pulled…so I ended up [going to a closer dental place…along with that came not as good service or you’d be waiting 2 h…”* *“…what I need, for instance, is access to an emergency room immediately if I’ve been hurt.”*
Economic	Affordability of care Economic stability Hiring diversity Sustainability Workforce development	Reallocate resources Hiring diversity	*“I do not feel like parents should have to pay for meal tickets and like, you know, to eat while [their] kids [are] staying there at the hospital. You know, they expect parents to be able to have the funds to go back and forth and then feed themselves”* *“The health care system seems more to me like a corporation because it seems like if you do not have health insurance, that you are not going to get the best treatment.”* *“…to have the opportunity to go to any health care center that specializes in said health concern. We need that world, do not we?”*
Policy	Equitable policy enforcement Power sharing	Redistribute power Close gaps between standards or policies and practice	*“…ears that are willing to hear the morality of it all…anybody that has any effect on budgets at the federal level… So, I guess that’s top-level politicians, business leaders.”*

The team compiled information from the literature scan, the St. Louis session, and the first Madison session into a digital collaboration tool to visualize the relations between the identified inductive concepts. Three overarching themes were identified to organize the health equity concepts: trust, power, and access. These themes guided our adaptations to the framework. Initial conversations yielded approximately 20 health equity benefits. At that point, the UW-Madison team met for multiple hours to review, discuss, and refine the list of indicators down to 14, with quotes that reflected or summarized those indicators. An additional series of meetings brought both campus teams together to reflect and refine, seeking to avoid overlap with existing benefits, to create discrete categories that were broad enough to allow for customization and operationalization, and to select the benefits that had the strongest evidence for inclusion across all groups consulted. This resulted in 10 proposed benefits for the TSBM.

We took those working benefit titles and definitions back to community members in Madison for feedback. Specifically, we asked if each the 10 benefits across the four TSBM domains was important, if it was clear, and if it made sense to them. CARDS members were united in expressing that all 10 benefits felt relevant and important for improving health equity. Many stressed how important the benefits were. They also shared that the benefits all seemed connected, noting their inter-relatedness. As for clarity, participants agreed that most of the benefit titles made sense and were easy to understand. The three exceptions were called “Trusted decision-making,” “Equitable systems and structures,” and “Policy engagement” at the time. CARDS members emphasized that decision-making first and foremost should be *shared*. They found “systems and structures” too vague, expressing that features of the *built environment* for health and healthcare – like transportation, location, accessibility – were most important after talking through examples, and that “engagement” in policy was too broad.

Participants found about half of the 10 definitions straightforward, and others unclear, remarking that while the titles made sense, many phrases in the definitions as presented were sometimes too academic, unnecessarily complex, or vague. Examples include *co-creation*, *dismantling structures*, *service inequities*, and *economic activities*. Throughout the course of discussions, CARDS members expressed sentiments that the definitions should “just say that then” after asking for more information. There were also discussions about the terms *historically underrepresented* and *underserved*. Reflecting larger concurrent societal-level struggles with myriad terms used to describe groups with less or no power, no consensus was reached in the listening sessions, though some made suggestions of using *minorities* or *minoritized*, or explicitly stating the groups to whom statements refer. In our conversations with community members, and as colleagues, we were reminded that these benefits will be operationalized within studies as variables. As an example, a single research study could not “provide access to health resources where all people need them.” Rather, a team or teams might collectively research best responses to a community need, and in aggregate many studies working together will create shifts that build Community Resource Access.

The importance of the previously identified overarching themes of trust, power, and access were confirmed by the community input from the second Madison session. The members highlighted the importance of trust, power, and access in addressing and increasing attention to health equity in the TSBM. This trust is between patients and providers as well as communities and researchers. Participants agreed enthusiastically when others said things like:

*“If you don’t have trust, the other benefits will never happen.”*

*“There are more ways to build trust than just explaining benefits and risks [to patients].”*

*“…trust builds when providers encourage patients to be part of the decision-making process.”*

*“Doesn't it take time to build trust? And usually in these research things, if you go in there, you don't know the researchers. They don't know you.”*

Different aspects of power were also central in the sessions. One participant mentioned that “*anybody that has any effect on budgets at the federal level*” (Table 1) needs to hear the session discussions, and multiple CARDS members pointed out that using superlatives in the benefits implies that somewhere, someone holds the power to decide. Phrases like delivering health services *to those who need them **most*** or *to the* ***most*** vulnerable populations do not imply universal agreement on who or where those communities or individuals are. Participants also expressed the need for the power that comes with options, to avoid, for example, hours of travel, waiting, or childcare and cost-prohibitive incidental expenses of healthcare like family expenses for room and board. Closely related to power is access. Beyond but not excluding usually cited barriers to health and healthcare like cost and proximity, fairer access to specialists and earlier access to innovative treatments are also important, for example.

### Equity benefits in the TSBM

Using all the feedback and knowledge generated through our discussions with community organizations and members, along with our previous work, we have developed 10 benefits. The new benefits, along with definitions are highlighted in Table 2, where the rightmost columns highlight the running themes.

**Table 2:** TSBM equity benefits.

Domains and benefits	Definitions	Trust	Power	Access
Clinical				
Clinical innovation access	Timely access to clinical advances for all	✓		✓
Patient-guided research	Research that engages patients throughout and aligns with patient priorities	✓	✓	
Shared decision-making	Interactions between providers and patients that are clear, understood, and create trust	✓	✓	
Community				
Community power & partnerships	Relationships between people, researchers, and providers built on power sharing	✓	✓	
Healthy built environment	Services, spaces, and places that support everyone’s well-being		✓	✓
Resource access	Access to health resources when and where all people need them		✓	✓
Economic				
Diverse healthcare workforce	Expanded opportunities for all people in healthcare and health research		✓	✓
Equitable healthcare economies	Broadened distribution of income and wealth in healthcare		✓	✓
Policy				
Community-guided policy	Community perspectives are clear, apparent, and drivers of the policymaking process	✓	✓	
Social justice through policy	Policies address, decrease, or erase health disparities and build social justice		✓	✓

New equity benefits in the Clinical domain are patient-centered, in both healthcare and research settings. Those in the Community domain focus on collaborations and increased support for all people and groups where they are needed. The new benefits in the Economic and Policy domains consider fair opportunities and inclusive policies and practices that address various types of existing disparities.

Finally, the equity additions are shown in context with all the TSBM benefits in Table 3. The new expanded TSBM framework now has 40 benefits spread across the four domains of clinical, community, economic, and policy impacts. Each domain now has three or four subdomains, that now include equity increases.

**Table 3:** Equity increases subdomains and benefits in context.

Clinical	Community	Economic	Policy
Equity increases Clinical innovation access Patient-guided research Shared decision-making	Equity increases Community power & partnerships Healthy built environment Resource access	Commercial products License agreements Non-profit or commercial entities Patents	Advisory activities Committee participation Expert testimony Scientific research reports
Procedures & guidelines Diagnostic procedures Investigative procedures Guidelines Therapeutic procedures	Health activities & products Community health services Consumer software Health education resources	Equity increases Diverse healthcare workforce Equitable healthcare economies	Equity increases Policy engagement Social justice through policy
Tools & products Biological factors & products Biomedical technology Drugs Equipment & supplies Software technologies	Health care characteristics Health care accessibility Health care delivery Health care quality Health promotion Disease prevention & reduction Life expectancy & quality of life Public health practices	Financial savings & benefits Cost effectiveness Cost savings Societal & financial cost of illness	Policies & legislation Legislation Policies Standards

## Discussion

To demonstrate and evaluate the translational impacts of science and research it is necessary to consider how research advancements affect opportunity, fairness, and justice – the cornerstone principles of equity. With equity comes trust, power, and access; we designed the new TSBM benefits to reflect these themes. Assisted by community organizers, patients, and societal representatives, we amended the TSBM to include 10 new benefits in Equity Increases domains. Those in the clinical domain focus on fair access, voices that are heard, and the active participation for patients in care, the planning of care, and new lines of research and discovery. The community benefits are advances that level the playing field between people – as patients, families, and community members – and providers and researchers. Inclusive representation, opportunity, and income distributions across roles in healthcare underlie the economic benefits. And policy efforts – both small “p” organizational and large “P” governmental policies – that demonstrably integrate perspectives from the communities they will impact and diminish barriers to health and well-being for all are highlighted in the policy domain.

Through our efforts to infuse considerations of equity into the TSBM, we also critically reviewed the original benefits to explore whether and how they could be understood through an equity lens. We found that multiple TSBM users had already done this through case studies of their own work. We also found many instances where issues of equity fit naturally in the definitions, longer descriptions, rationales, and examples and can use these as opportunities to update the framework. Along with the new benefits, these updates will make a renewed TSBM itself more accessible and applicable in more areas of research, evaluation, policy, and practice.

Issues of power and trust are not new ideas when thinking about equity. It is perhaps unsurprising that they rose as overarching themes of our efforts, and community member input in particular. This could be perceived on one hand as affirming of our efforts, and on the other as issues that bear repeating. Power has many faces – political, social, economic – and comes with control over rules and other institutions and practices, both formal and informal. The new TSBM benefits focus on sharing these types of power among all people, and highlighting when efforts are successful. Power, or the lack of it, is also found in more everyday aspects of life, like access, choices, and opportunities. The new benefits also draw attention to points when people get more of these.

Power goes hand-in-hand with trust. Built over time, trust in patient-provider and community-researcher relationships comes with more than just explaining benefits and risks of treatment or handing out pamphlets, decision aids, or financial incentives. It comes with sustained engagement, across clinical visits and providers, and long-term, mutually beneficial community-researcher partnerships. Trust develops when patients and people are listened to, heard, and believed, with awareness that their experiences and perspectives matter, and that all these have mattered in previous interactions and play a part in shaping their future. The new TSBM benefits also reflect this, and as in instances where power is fairly distributed, serve as mechanisms to emphasize when trust is mutually shared.

Though the primary users of the TSBM are scientists and researchers, the motivation driving it and its *translational* rationale imply the need for accessibility for various audiences. While they may seem simple, changes to wording and purposive definitions serve to broaden the framework’s accessibility and understanding for more people, including our own team. Following up with CARDS members after the initial listening sessions and initial development of the new benefits to get more thoughts and reactions was crucial to this process and cannot be underestimated. We are tremendously appreciative of the time and input all the community members gave to the project and look forward to sharing back the updated TSBM with them. In addition, planning for, using, and demonstrating the new TSBM benefits will require more input from evaluators in CTSA hubs, patients, people, and communities throughout the research process. Concepts like power and trust are not easily inferred secondhand and necessitate evidence, testimonials, and stories from the people who feel impacted in a positive way. This will require training for scientists and researchers beyond those whose work focuses mainly or explicitly on issues of equity.

In addition to sharing updates with partners and gathering feedback from researchers, next steps include continuing to update the TSBM by developing longer descriptions and examples for the new benefits and refreshing the original ones with new examples. We are actively working to flesh out the new benefits to make them more distinct and ensure that each category is clearly defined. This ongoing process, which includes gathering feedback from stakeholders and deeply reviewing existing benefits, aims to clarify the specific benefits being addressed and to minimize any ambiguity. We have developed and continue to refine language for the rationale behind each new benefit to further explain why each is an important impact of translational science and research. The rationale, along with a detailed description that includes examples, guidance for finding and collecting information to demonstrate each benefit, and a curated list of relevant resources and publications, will complete this work and mirror the supportive elements provided for the original 30 benefits on the TSBM website (22). As the new health equity benefits are integrated into web-based TSBM tools, there will be more detail regarding each of the benefits and how they are distinct. This process has not been completed and therefore not included in this paper.

This work has several implications for research, evaluation, and practice. The TSBM framework has been in use since 2018, and the toolkit since 2021. The number, diversity, geography, and substantive areas of uses and users continue to grow. The additional focus of health equity and 10 benefits that explicitly centralize how the impact of science and research can improve well-being for all people and communities expands the relevance and application of the TSBM. Rather than an “evaluation checklist” for impacts of science and research, the TSBM offers a “menu of potential benefits” for communities and society. Increases in its reach and visibility can inspire those in research to integrate health equity considerations earlier in their research planning and inspire those in clinical practice to share successful strategies that result in mutual trust and openly shared decision-making. The updated TSBM can also encourage its adaptation to new arenas and further facilitate its use in and beyond educational, health, healthcare, and public health programs and institutions. Work and evaluation in physical and social sciences, social work and public policy, political-, social-, economic-, and community-based programming along with international development efforts can more readily take advantage of the TSBM to systematically design, document, demonstrate, and disseminate progress and downstream impacts for individuals, communities, and society.

## Data Availability

The raw data supporting the conclusions of this article will be made available by the authors, without undue reservation.
