# Supplementary material for: Who benefits? Health equity and the Translational Science Benefits Model
Source: Front Public Health. 2025 Apr 3;13:1565248. doi: 10.3389/fpubh.2025.1565248 (PMC12003293; doi:10.3389/fpubh.2025.1565248)
Supplement: Supplementary file 1 [file Data_Sheet_1.docx]

**Appendix A. Sources for full review from the literature scan.**

Aguilar efren, Perrigo JL, Pereira N, Russ SA, Bader JL, Halfon N. Unveiling early childhood health inequities by age five through the national neighborhood equity index and the early development instrument. SSM - Popul Health. 2024;25:101553. doi:10.1016/j.ssmph.2023.101553

Allan R, McCann L, Johnson L, Dyson M, Ford J. A systematic review of ‘equity-focused’ game-based learning in the teaching of health staff. Public Health Pract. 2023;7:100462. doi:10.1016/j.puhip.2023.100462

Brownson RC, Kumanyika SK, Kreuter MW, Haire-Joshu D. Implementation science should give higher priority to health equity. Implement Sci IS. 2021;16:28. doi:10.1186/s13012-021-01097-0

Gómez CA, Kleinman DV, Pronk N, et al. Addressing Health Equity and Social Determinants of Health Through Healthy People 2030. J Public Health Manag Pract. 2021;27(6):S249-S257. doi:10.1097/PHH.0000000000001297

Howard S, Lefler L, Jiang Y. Using the Community Action Poverty Simulation to Accelerate Learning About Health Equity. J Nurse Pract. 2023;19(10):104790. doi:10.1016/j.nurpra.2023.104790

Krahn GL, Fox MH. Health Disparities of Adults with Intellectual Disabilities: What Do We Know? What Do We Do? J Appl Res Intellect Disabil JARID. 2014;27(5):431-446. doi:10.1111/jar.12067

Mohottige D, Diamantidis CJ, Norris KC, Boulware LE. Racism and Kidney Health: Turning Equity Into a Reality. Am J Kidney Dis Off J Natl Kidney Found. 2021;77(6):951-962. doi:10.1053/j.ajkd.2021.01.010

Nichols LM, Taylor LA. Social Determinants As Public Goods: A New Approach To Financing Key Investments In Healthy Communities. Health Aff Proj Hope. 2018;37(8):1223-1230. doi:10.1377/hlthaff.2018.0039

Pedrana L, Pamponet M, Walker R, Costa F, Rasella D. Scoping review: national monitoring frameworks for social determinants of health and health equity. Glob Health Action. 2016;9:10.3402/gha.v9.28831. doi:10.3402/gha.v9.28831

Salter K, Salvaterra R, Antonello D, et al. Organizational level indicators to address health equity work in local public health agencies: A scoping review. Can J Public Health Rev Can Santé Publique. 2017;108(3):e306-e313. doi:10.17269/CJPH.108.5889

Taylor JY, Barcelona V, Magny-Normilus C, et al. A Roadmap for Social Determinants of Health and Biological Nursing Research in the NINR 2022–2026 Strategic Plan: Optimizing Health and Advancing Health Equity using Anti-Racist Framing. Nurs Outlook. 2023;71(6):102059. doi:10.1016/j.outlook.2023.102059

Wilf-Miron R, Avni S, Valinsky L, et al. Developing a National Set of Health Equity Indicators Using a Consensus Building Process. Int J Health Policy Manag. Published online June 23, 2021:1. doi:10.34172/ijhpm.2021.54

Wong ST, Browne AJ, Varcoe C, et al. Development of Health Equity Indicators in Primary Health Care Organizations Using a Modified Delphi. PLoS ONE. 2014;9(12):e114563. doi:10.1371/journal.pone.0114563

Woodward EN, Singh RS, Ndebele-Ngwenya P, Melgar Castillo A, Dickson KS, Kirchner JE. A more practical guide to incorporating health equity domains in implementation determinant frameworks. Implement Sci Commun. 2021;2(1):61. doi:10.1186/s43058-021-00146-5

Yousefi Nooraie R, Kwan BM, Cohn E, et al. Advancing health equity through CTSA programs: Opportunities for interaction between health equity, dissemination and implementation, and translational science. J Clin Transl Sci. 4(3):168-175. doi:10.1017/cts.2020.10

**Appendix B. Supplementary quotations from listening session.**

| “…so, people I feel like aren't able to access [transportation]. And there are people that miss appointments simply because it's just like it's too much of a struggle to have to load up your two kids on a bus, you know? And I've been in predicaments where I'm like, I'm not going to ride the bus an hour with my two kids. I rather just get this appointment, unfortunately, because it's too much.” |
| --- |
| “I have a friend that daughter has like a couple of health issues, so she has to have a children's hospital frequently. And like, I don't feel like parents should have to pay for meal tickets and like, you know, to eat while they're kids, you know, and and staying there at the hospital. You know, they expect parents to be able to have the funds to go back and forth and then the feed themselves.” |
| “So, like with like with dental care…they wanted me to go travel all the way to Milwaukee to get my tooth pulled, which wasn't convenient for me. And so I ended up having to go switch to another closer dental place. But the quality of service was so bad and, you know, they took off seeing insurance people and, you know, unfortunately, along with that came not as good service or you'd be waiting 2 hours for one thing… You know, so putting your health at bay because I'm, you know, because of quality issues.” |
| **“**When it comes to benefits, personal benefits, the first thing I need is, is real life benefits. Not out there, philosophical benefits being discussed in the community. So, what I need, for instance, is access to an emergency room immediately if I've been hurt. I'm a bicycle rider here. I've been hit twice. And in real life, that's what I need. I have pretty good access to emergency rooms now, due to a couple of good programs. But there's ever so many people who don't have or have to travel too far or this or that or the other. I say make it available to all. And I think also people of lower income are perhaps more likely to get hurt or need facilities like that. Well, let's make it available.” |
| “…it's the accessibility and affordability… I mean, insurance seems to be, you know, a huge problem for things being equal for people in general. I mean, I have insurance and yet there are you know. I'm a senior I ran into, you know, the donut hole this year. I had a lot of medical expenses this year. And all of a sudden, I'm seeing, you know, I don't know how people can even afford these drugs that don't have insurance. And just trying to wade through the insurance feels like it makes things inaccessible.” |
| “I'm very lucky to have access to health care right now. But I did notice like on one occasion, I've had like, a pharmacist tell me that like, basically something was like too expensive for the company to do or the business to do. So, they would choose not to, I don't know, take it as liability.” |
| “I just want to say the health care system seems more to me like a corporation because it seems like if you don't have health insurance, that you're not going to get the best treatment when you go to the hospital or a clinic. I've heard of that happening to people where they didn't have insurance, and they didn't get that. They was patched up and sent home when they had a more urgent health scare…. I always just tell my kids when they were in high school, always keep your health care card because you know something happens. They'll be able to look in your wallet and get your health care card and see that you have insurance so that you can get the best treatment.” |
| “Again, like within our community, it's hard for people to trust health care providers. It's hard for people to also understand what they're talking about. It's hard for people to know even what some of the symptoms or different things that they are dealing with looks like. And so, I think we have to start with that because… some people aren't even going to the doctor, some people aren't even going to these places. Some people aren't even, you know, attempting to try because they don't know, they don't trust or they just don't understand.” |
| “… with a lot of African American women, you know, we've been gaslighted, you know, we all feel like I have. I told my primary care, look, this is what's going on, you know. You know your body, you know your body. You know that something isn't right…. I appreciate the research, but your doctors, your primary care, they have to listen and stuff, too. They don't listen to when you telling them about certain things you want to do…. I do appreciate research for different things that, you know, researchers are doing to find different ways of finding out what's curable, what's not…. But also, you know, your doctors, they have to listen to you and they really, truly don't.” |
| “…when it comes to treatment itself, I see a lot of places having the three month, four months, six month wait just to get like specialty appointments…. even if you have like the SSA, even if you have the ability to like talk to someone, actually getting the care that you need is probably going to be coming like months down the line.” |
| “I feel like within a reasonable amount of time, like if someone is having a cavity that's affecting them, say I'm like, we can get you in in three months is like—am I even gonna be alive in three months? Like, I don't know if that's going to be salvageable. So, some stuff is obviously I can wait, but like, I just have noticed a lot of things that you see that I would think would get taken care of a lot sooner people have to wait for.” |
| “…just getting information like about studies out there. People that could benefit from it is a good thing…. So, I think that's like my biggest thing is just like hearing about [research] and yeah just be able to benefit from like the research.” |
| “…just listen and just give everyone the health care that they need, the researchers just put things out there, you know, the research that they make it available for the public to know, the research they've been doing and the benefits of the research” |
| “A research benefit would be to prevent further decline in health. So, it wouldn't just be dealing with things getting worse and worse, but actually preventive treatments—to have the opportunity to go to any health care center that specializes in said health concern. We need that world, don't we?” |
| “…for it to have effect on not just my life but everybody else's life is this message has to go out to eyes that are willing to see the reports and read the reports, ears that are willing to hear the morality of it all. [The people who should hear this are] anybody that has any effect on budgets at the federal level. For starters, decision makers who make the decisions on funding the priorities. So, I guess that's top-level politicians, business leaders.” |
| “[People would] probably be able to get *true treatment* instead of just being given pills. You know they're quick to have the doctors just throw out medication or just give them some medicine.” |
| “They want to give you all this medication and it's all these side effects and it's just like putting a Band-Aid on, you know? So, you're going to have to take this medication and then something else can go wrong, so you’re gonna need another medication.” |
| “…doctors rarely offer holistic or at home approaches and remedies. Lots of money goes to the pharmaceutical industry. People might actually want to go see their provider [if more options were offered]” |
